# Stress Monitoring in Pandemic Screening: Insights from GSR Sensor and Machine Learning Analysis

**DOI:** 10.3390/bios15010014

**Published:** 2025-01-02

**Authors:** Antonios Georgas, Anna Panagiotakopoulou, Grigorios Bitsikas, Katerina Vlantoni, Angelo Ferraro, Evangelos Hristoforou

**Affiliations:** 1 Laboratory of Electronic Sensors, National Technical University of Athens, 15772 Athens, Greece; annapan@biomed.ntua.gr (A.P.); el16693@mail.ntua.gr (G.B.); an.ferraro2@gmail.com (A.F.); hristoforou@ece.ntua.gr (E.H.); 2 Department of History and Philosophy of Science, School of Science, National and Kapodistrian University of Athens, 15771 Athens, Greece; kvlantoni@phs.uoa.gr

**Keywords:** stress monitoring, COVID-19 screening, galvanic skin response (GSR), machine learning analysis, biosensors, classification algorithms, physiological stress indicators

## Abstract

This study investigates the impact of patient stress on COVID-19 screening. An attempt was made to measure the level of anxiety of individuals undertaking rapid tests for SARS-CoV-2. To this end, a galvanic skin response (GSR) sensor that was connected to a microcontroller was used to record the individual stress levels. GSR data were collected from 51 individuals at SARS-CoV-2 testing sites. The recorded data were then compared with theoretical estimates to draw insights into stress patterns. Machine learning analysis was applied for the optimization of the sensor results. Classification algorithms allowed the automatic reading of the sensor results and individual identification as “stressed” or “not stressed”. The findings confirmed the initial hypothesis that there was a significant increase in stress levels during the rapid test. This observation is critical, as heightened anxiety may influence a patient’s willingness to participate in screening procedures, potentially reducing the effectiveness of public health screening strategies.

## 1. Introduction

It is a well-established fact that humans are susceptible to a wide variety of viruses, but the viruses themselves have varying impacts on populations. Some are relatively harmless, while others can cause serious pandemics that lead to widespread illness and significant loss of life. The 2020 SARS-CoV-2 pandemic has, to date, been responsible for millions of deaths and enormous disruptions to global commerce, education, and societal norms [1]. The virus mainly spreads through respiratory droplets and contact with contaminated surfaces. Testing and population screening have been key strategies in the attempt to control the spread of the virus, and governments all over the world have distributed huge numbers of diagnostic tests. Yet, while effective tests to identify infections are at hand, the public commonly does not understand how these tests work, which can increase anxiety and stress during the testing process.

Medical screening refers to preventing public health problems, such as an ongoing viral epidemic, by detecting early infections and providing epidemiological data on the rate of infection of a certain population group. Screening can be applied to high-risk groups or large populations, regardless of whether they exhibit symptoms (asymptomatic carriers), to permit early treatment and to confine the spread of the virus. In contrast, testing for diagnostic purposes is directed to individuals with symptoms and has clinical value for medical treatment. The main difference between screening and testing is that screening targets asymptomatic individuals and is generally cheaper, whereas testing targets symptomatic individuals and is more precise. Screening is designed to be easily acceptable to the public and functions as a preventive measure, albeit with a higher chance of false positive or false negative results compared to testing [2]. While testing is focused on providing accurate results and is part of the treatment process, screening serves as a broader public health strategy. This distinction is crucial because screening, despite its lower sensitivity and specificity, is essential to identifying at-risk individuals early.

Galvanic skin response (GSR) refers to the variation in the skin’s conductance following a stimulus that induces emotional arousal. GSR results from the autonomous activation of the sweat glands in the skin, reflecting the fluctuation in the electrical properties of the skin. GSR activity is typically measured in micro-Siemens (μS) and normal human skin conductivity (GSR) ranges from 1 to 20 (µS) [3,4].

GSR provides insights into emotional arousal but not the type of emotion. During exposure to stimuli that cause emotional arousal, bodily processes are automatically activated: the heart beats faster, the pulse increases, sweat secretion increases, and ultimately, GSR increases. Specifically, when a subject is stressed, nervous, scared, excited, confused, or surprised—each time a stimulus emotionally arouses them, in any way, positive or negative—the electrical conductance of their skin changes.

The reason GSR is such a valuable biometric signal in assessing emotional behavior is that it leverages unconscious behavior not under cognitive control. Skin conductance (EDA) is exclusively regulated by autonomic sympathetic activity, which drives bodily processes, cognitive and emotional states, and knowledge on an entirely subconscious level. One cannot consciously control the level of skin conductance. This fact makes GSR the perfect indicator of emotional arousal, offering indispensable insights into an individual’s physiological and psychological processes [5].

Based solely on GSR, one cannot deduce whether the arousal is due to positive or negative stimulus content. Both positive and negative stimuli can lead to increased arousal, triggering GSR peaks. Regardless of the emotion being positive or negative, GSR may be identical. While GSR is an ideal measure for monitoring emotional arousal, it cannot reveal the quality of the emotions. The true power of GSR unfolds when combined with other data sources (Pupil Dilation (PD), Eye Tracking (ET), Heart Rate (HR), hyperventilation) to portray a complete picture of emotional behavior [6].

Recent advancements in machine learning have enabled more precise analysis of physiological data [7], including galvanic skin response (GSR), which is commonly used to assess stress levels [8,9,10]. By leveraging machine learning algorithms, it is possible to identify patterns and classify stress states with greater accuracy than traditional methods. Machine learning methods are particularly effective in analyzing complex, nonlinear, and non-stationary datasets, making them highly applicable to stress evaluation studies. These methods allow for the identification of hidden patterns and correlations within large datasets, even under variable conditions. In medical diagnostics, machine learning has been successfully employed in areas such as early disease detection, personalized treatment plans, and real-time patient monitoring [11,12]. This study explores the application of machine learning techniques to enhance the analysis of GSR data, aiming to improve the detection of stress responses in varied contexts.

This manuscript examines how the GSR behaves in response to intense stimulus that causes surprise, stress, or emotional arousal. Measurements were taken with a sensor that detects the GSR. GSR measurements were made by visiting COVID-19 testing sites that performed rapid tests and using a GSR sensor, connected to an Arduino UNO microcontroller, which in turn was connected to a computer [13]. Measurements results, after processing and analysis, were compared with the theoretical estimates to draw insights into stress patterns. Machine learning analysis was applied for the optimization of the sensor results. Classification algorithms allowed the automatic reading of the sensor results and individual identification as “stressed” or “not stressed”. Finally, it was examined whether the obtained results confirm the initial hypothesis that “there is an increase in the levels of anxiety experienced by the subject during the rapid test for the COVID-19 pandemic”.

## 2. Materials and Methods

### 2.1. Galvanic Skin Response Sensor

A GSR sensor (Seeed Technology Co., Ltd., Shenzhen, China) connected to an Arduino UNO microcontroller and a computer were used. The GSR sensor measures electrical conductivity (EDA) produced by sweat glands. Strong–intense emotion or stress leads to an increase in the galvanic skin response (GSR). This sensor works with an Arduino UNO module to record, store and transmit the measurements to a computer.

**Figure 1:**
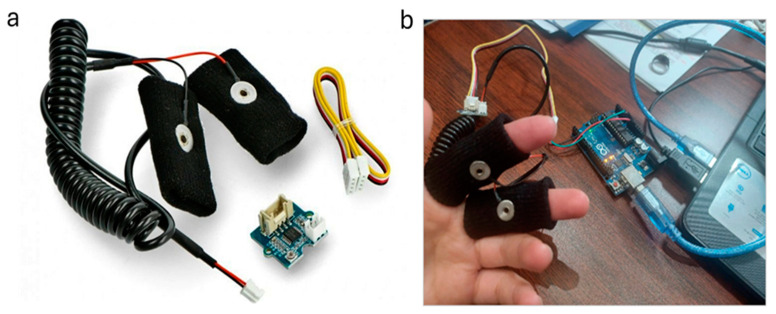
(**a**) The GSR sensor; (**b**) individual wearing the GSR contacts.

The GSR sensor was connected to the Arduino UNO as illustrated in Figure 1:

- The Vcc port of the GSR sensor was connected to the 5 V port of the Arduino (red cable) and the ground port (Ground or GND) of the sensor with the corresponding one of the Arduino (black cable).
- A wire connected the signal port (SIG) of the sensor to the A0 port of the Arduino (green wire), where the output was received.
- The Arduino UNO module was in turn connected to a computer, through which the module was programmed and the measurements were visualized.

### 2.2. Protocol Used to Conduct the Experiment

Data were collected via the GSR sensor connected to an Arduino UNO and a PC from 51 subjects who took a rapid test for COVID-19 in COVID-19 testing sites such as pharmacies, during July of 2022. Data were obtained in real-time, non-invasively, by measurements taken from two fingers of the patient. Each experiment lasted approximately 1–2 min on average. The duration varied from user to user, as the process of taking the test could be quicker for some and slower for others. During this time, GSR data are recorded at intervals of every 2 s, resulting in multiple data points for analysis.

The measurement process begins once the software command “Serial Monitor” is given from the computer, which starts the measurement. During the 1–2 min of experiment, the data were displayed via the Arduino IDE on the computer screen. The measurement did not have a predetermined duration; it simply ended when the subject removed the sensor.

All people involved in the research were informed about the use of the collected data and provided written consent to both data collection and data publication. No personal data have been recorded and all measurements have been stored with anonymous codes.

### 2.3. Data Processing and Unit Conversion

For this specific application, the code used to receive and record the measurements was developed in the Wiring language. Development was carried out in the Arduino IDE programming environment.

Although the output of the GSR sensor is an electrical voltage (Volts), the Arduino records the measurements in Arduino Units (AU). These units are defined as (1/1024) of the Arduino UNO’s maximum supply voltage (here 5 V).

The values in AU should be converted to Volts to have a physical meaning.

For example, the value 512 AU corresponds to

(1) $$512\;AU\;\cdot \;\left(\frac{5}{1024}\right)\cdot \left(\frac{V}{AU}\right)\;=\;2.5\;V$$

Given that the most used unit for describing GSR signals in the literature is microSiemens (μS), and based on the definition of Siemens as the reciprocal of the unit of ohmic resistance (Ω, Ohm), the following steps were taken:

- Step 1: Calculation of the voltage Vx measured by the sensor by converting Arduino Units (AU) to Volts. For this, the conversion factor λ was used:

(2) $$\lambda \;=\;\frac{5}{1024}\;=\;0.0048828125\;≅\;4.9\;\frac{mV}{AU}$$

**Figure 2:**
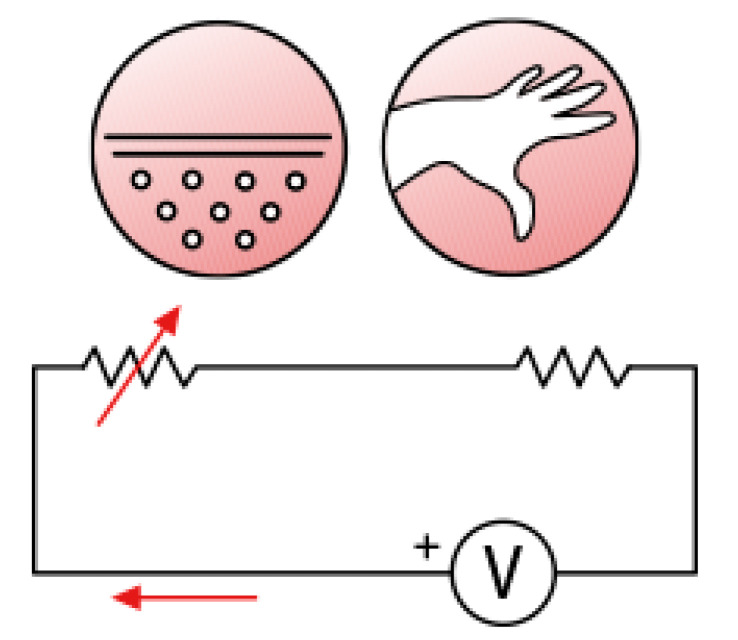
The equivalent electrical circuit.

- Step 2: Calculation of the current (I) through the GSR sensor (in μA). Based on the equivalent electrical circuit topology that is illustrated in Figure 2

(3) V=I (R+X)

(4) Vx=I×X

where

V = supply voltage (5 V);

R = equivalent sensor resistance (fixed at 500 KΩ);

X = the unknown measured resistance of the subject’s hand;

I = sensor current (varies with X);

Vx = voltage drop across the resistance X.

By solving this system, we obtain

(5) I = (V−Vx)/R

(6) X = Vx/I 

- Step 3: Calculation of the conductance Y, where Y = 1/X 

The current I is calculated using the formula

(7) I = (5 − Vx)/(500 KΩ) 

The result is multiplied by 10^6^ to convert from Amperes (A) to microAmperes (μA). Having found the current (I) (in μA) and the voltage Vx (in Volts) across the unknown resistance X at each measurement moment, the unknown resistance X can be calculated. The result is presented in kΩ (kiloohms).

Finally, the calculated resistance X is inverted to obtain the conductance Y = 1/X, which is then presented in microSiemens (μS). To visualize the results, the data were rendered through graphical representations of the GSR (in μS) as a function of time (in seconds).

### 2.4. Data Visualization and Analysis

**Figure 3:**
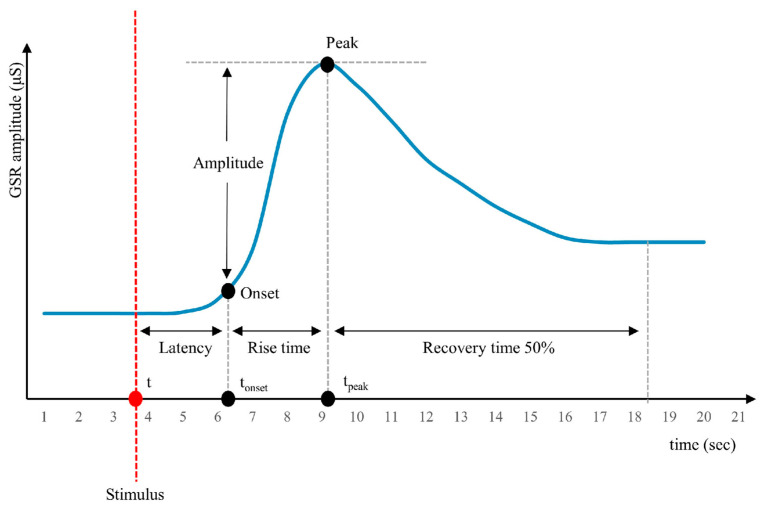
Galvanic skin response (GSR) signal characteristics following a stimulus. The red dot and vertical dashed line indicate the stimulus time (t). Key parameters include latency (time from stimulus to response onset), rise time (time from onset to peak), amplitude (peak GSR value minus baseline), and recovery time (time to reach 50% of the peak amplitude after the peak) [14].

In the GSR signal there are some basic characteristics that are encountered when measuring with the sensor on a person who is stressed or generally in a state of cognitive arousal. These basic characteristics are Latency, Peak Amplitude, Rise Time and Recovery Time, as illustrated in Figure 3 [14].

1. Latency: The duration from the onset of the stimulus to the onset of the phasic burst. Typically, ER-SCRs (Event-Related Skin Conductance Responses) appear 1–5 s after the onset of the stimulus. The onset is generally defined at the point in time where the GSR curve exceeds a minimum amplitude criterion (0.01 or 0.05 μS, respectively). GSR changes that occur before this period are typically defined as NS-SCRs (Non-Specific Skin Conductance Responses) and are not considered to be generated by experimental manipulation.
2. Peak amplitude: The amplitude difference between the onset and the peak.
3. Rise time: The duration from the onset to the peak.
4. Recovery time: The duration from the peak to the 50% recovery of the initial tonic GSR. While the onset of an SCR can be quite abrupt, the recovery is usually more gradual, resulting in longer recovery times.

The representation in Figure 3 is a simplification, as the raw GSR signal is not completely flat before or after a peak. Instead, it varies due to individual differences in tonic GSR levels or noise from movement artifacts or respiration. Analyzing the recovery time after peaks is usually much more challenging compared to analyzing measurements related to onset and peak.

Filtering: A filter can ’smooth’ the GSR curve to remove the tonal component of the signal unrelated to excitation or high amplitude ’spikes’ created by motion. A median filter achieves this, leaving only the phasic signal. A basic median filter can be applied in three steps: (1) Going through the data per sample. (2) For each sample, the intermediate GSR score of the surrounding samples is calculated based on a +/−4 s time interval centered on the current sample. (3) The mean value is subtracted from the current sample. The result is the phasic data.

Onset and peak detection: algorithms and procedures can be used to automatically detect and report the onsets and peaks in a record and find the maximum GSR value.

Calculation of the GSR peak amplitude: an important indicator of GSR data analysis is the GSR peak amplitude, which is equal to the amplitude at the peak minus the amplitude at the onset.

### 2.5. Training of Machine Learning Algorithms for GSR Data Analysis

Machine learning algorithms were employed to analyze and classify GSR data collected from subjects undergoing a COVID-19 rapid test, as well as from pre-existing datasets. The goal was to differentiate between stressed and relaxed states based on GSR signals.

#### 2.5.1. Datasets

Two datasets were used for training and testing purposes:

- YAAD GSR Dataset: The Young Adult’s Affective Data (YAAD) dataset includes GSR and ECG data collected from 25 participants, serving as the primary training set. This dataset contains normalized GSR signals, making it suitable for supervised machine learning tasks [15].
- COVID-19 and Esports Testing Data: GSR data were collected during COVID-19 diagnostic testing procedures and from the “Collection and validation of psychophysiological data from professional and amateur players: a multimodal esports dataset”, which includes GSR data from 10 players over 22 League of Legends matches [16].

These datasets were used as the test sets for the model validation.

#### 2.5.2. Data Preprocessing

The raw GSR data from both datasets required preprocessing before they could be used for model training. This included the following:

- Normalization: The GSR values were normalized to a range between 0 and 1. This was necessary to handle variations in measurement scales, improve model stability, convergence, and fairness in comparisons between features.
- Feature Extraction: Key features such as GSR peak amplitude, GSR peak width, and phasic components were extracted to serve as input for the machine learning models. The phasic GSR signals were computed by subtracting the local average (over a 4 s window) from the original GSR signal to isolate rapid changes associated with stress responses.

#### 2.5.3. Model Selection and Training

Two machine learning models were trained for GSR data analysis:

1. K-Means Clustering: This unsupervised learning algorithm was employed to categorize the GSR data into clusters based on the level of stress. The number of clusters was set to 2, representing “stressed” and “relaxed” states. The GSR peak amplitudes and phasic signals were used as inputs for the clustering process.
  - Training Process: The K-means algorithm was applied to the training data from the YAAD dataset. It iteratively assigned each data point to one of two clusters based on the Euclidean distance between the point and the cluster centroid.
  - Model Evaluation: The clustering results were visually inspected, and the clusters were labeled as “stressed” and “relaxed” based on the GSR data patterns.
2. Support Vector Machine (SVM): For supervised learning, an SVM algorithm was used to classify subjects into stressed or relaxed categories based on labeled GSR data. The SVM model was trained on the YAAD and esports datasets, where the GSR peak amplitude and phasic component were used as input features. The goal was to learn a decision boundary that separates the two classes effectively.
  - Training Process: The SVM algorithm was trained using both a linear kernel and the Radial Basis Function (RBF) kernel to handle non-linear data separations. The hyperparameters, including the regularization parameter C and the kernel coefficient gamma, were fine-tuned using grid search with cross-validation. Both kernels were compared to evaluate their performance.
  - Model Testing: The trained SVM model was tested on the GSR measurements of the individuals undertaking a COVID-19 rapid test. The classification accuracy was evaluated by comparing the predicted stress labels with manually annotated stress states. Stress was manually annotated by observing the peak values of the GSR signal amplitude and their timing relative to known stress-inducing events (e.g., the start of the COVID-19 rapid test). The classification scores obtained using the linear kernel were compared with those of the RBF kernel to assess their respective effectiveness.

#### 2.5.4. Cross-Validation and Hyperparameter Tuning

For both models, 5-fold cross-validation was performed on the training set to avoid overfitting and ensure generalizability. The dataset was randomly split into five subsets, and the models were trained and validated across all combinations of these subsets. Hyperparameters were optimized using grid search for SVM, while the K-means model parameters were manually tuned based on clustering performance.

#### 2.5.5. Evaluation Metrics

To evaluate the performance of the SVM model, two key metrics were used: accuracy score and F1 score.

- Accuracy Score: This metric compares the expected labels of the samples with the classification results generated by the SVM model. The result is expressed as the percentage of correctly classified samples, showing how closely the predicted labels match the actual labels.
- F1 Score: The F1 score combines both precision and recall into a single metric by calculating their harmonic mean. This metric is specifically useful for binary classification problems, which in this study involved categorizing the subjects into two classes: stressed (positive class) and relaxed (negative class).

Precision measures the ratio of true positive classifications (correctly identified stressed subjects) to the total number of samples classified as stressed (both true and false positives). Recall measures the ratio of true positive classifications to the total number of actual stressed subjects (both correctly and incorrectly classified).

The formulas used to calculate precision, recall, and the F1 score are as follows:

(8) $$Precision\left(P\right)=\frac{TP}{TP+FP}$$

(9) $$Recall\left(R\right)=\frac{TP}{TP+FN}$$

(10) $$F1=\frac{2\times P\times R}{P+R}$$

where

- TP is the number of samples correctly assigned to the positive class (True Positive);
- FP is the number of samples incorrectly assigned to the positive class (False Positive);
- FN is the number of samples incorrectly assigned to the negative class (False Negative).

## 3. Results

### 3.1. Graphical Representation and Analysis of GSR Responses

The GSR measurements from 51 subjects during COVID-19 rapid testing were divided into three main categories:

- Category 1 includes cases where GSR peaks were observed, indicating that the subject experienced stress or anxiety during the rapid test. This stress could be related to the testing procedure itself, concerns about the test results, or the potential impact of a positive diagnosis on personal and professional obligations.
- Category 2, observed less frequently, refers to subjects who exhibited no significant changes in GSR values during the test, suggesting they were in a calm, relaxed state.
- Category 3, the rarest, accounts for instances of experimental error, where the GSR values fell outside the expected range.

The criteria used to classify the measurements were as follows:

- Pattern analysis: We first examined whether the GSR-time graph followed the typical pattern of “Latency–Peak Amplitude–Rise Time–Recovery Time”, as previously described.
- Magnitude comparison: We then assessed whether the GSR magnitude during the “Latency” phase differed significantly from the “Peak” phase.
- Peak count: Lastly, the number of GSR peaks was measured. If no discernible peaks were observed compared to the average values, the individual was considered to be at rest.

**Figure 4:**
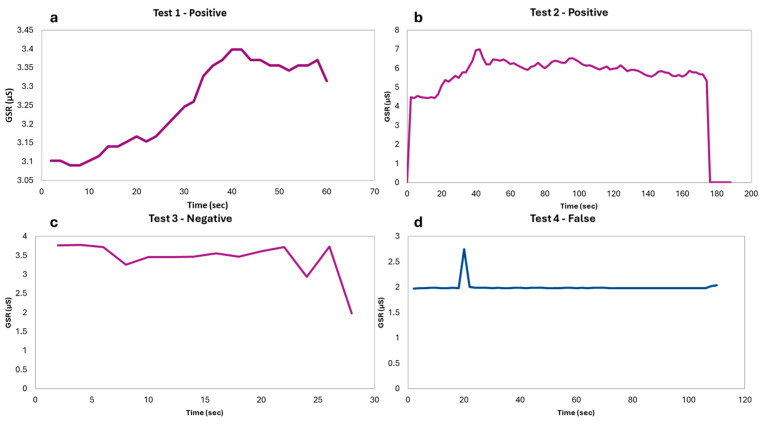
GSR response of four individuals; (**a**) stressed response (1); (**b**) stressed response (2); (**c**) not stressed response; (**d**) invalid measurement.

Figure 4 illustrates the GSR responses of four subjects during COVID-19 rapid testing. The tests are categorized as follows:

Test 1 and Test 2 (Positive): Both tests show distinct GSR peaks, with an overall increase in voltage levels as stress levels rise. In Test 1, the maximum GSR value reached X_max_ = 3.40 μS at t = 40 s. Following this peak, the GSR values begin to decrease, though the rate of decrease is slower than the rate of increase, with some local fluctuations. The latency period (time between stimulus and the onset of the phasic burst) is about 5–7 s. The peak amplitude is approximately 0.31μS, while the rise time is about 33 s, from the start to the peak. The recovery time cannot be calculated fully, as the measurement was interrupted before returning to baseline, but a slower recovery rate is observed, aligning with theoretical expectations. In Test 2, the ‘Latency–Peak–Recovery’ triptych is clearly observed. The latency period lasts approximately 20 s, with a peak amplitude of 2.51 μS (calculated as 6.99–4.48 μS). The rise time is measured at 22 s (T_rise_ = 42–20 s). Throughout the test, absolute GSR values remain consistently high. As in Test 1, the recovery time cannot be fully determined due to incomplete data; however, it exceeds 80 s (Δt = 174–94 s). This observation aligns with theoretical predictions, where recovery time is typically longer than the rise time.

Test 3 (Negative): Here, no significant change in GSR values was observed, indicating a state of calm or minimal stress during the rapid test. The subject exhibited no noticeable peaks, confirming a lack of stress response.

Test 4 (Invalid): This test represents an experimental error, where improper contact between the subject’s fingers and the GSR sensor led to invalid measurements. The constant value of 1.99 μS likely corresponds to the air conductivity, emphasizing the importance of proper sensor placement for accurate data collection.

After the GSR test, 36 out of 51 individuals, that is 71%, were listed as “stressed” during the SARS-CoV-2 rapid test. In total, 12 out of 51, that is 24%, were listed as “not stressed”, as there was no particular change in GSR values during the rapid test and therefore they were in a rest state. For the last three individuals, that is about 6%, no valid measurements were performed, due to some experimental error (e.g., poor electrode contact on the skin).

**Figure 5:**
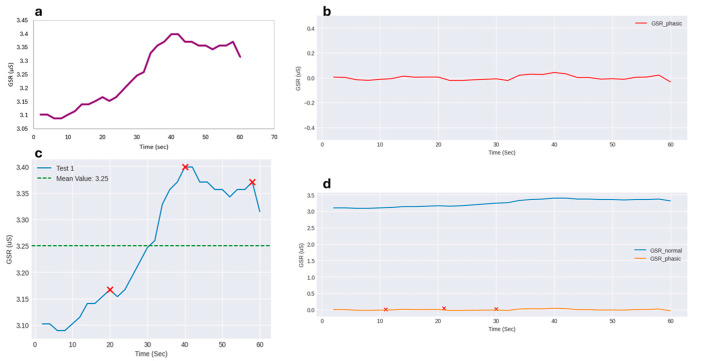
GSR signal visualizations; (**a**) raw GSR signal; (**b**) phasic GSR signal; (**c**) GSR peaks and mean value; (**d**) combined GSR and phasic signal.

Figure 5 shows four distinct types of GSR signal visualizations, each derived through different methods to highlight various aspects of the subject’s physiological response during the rapid test:

- (a) Raw GSR Signal: This graph represents the raw GSR signal, obtained by plotting the subject’s conductance (in µS) against time (in seconds). The data were processed in LibreOffice Calc, where the Y-axis corresponds to GSR values and the X-axis to time. This visualization falls into the first category of graphs and offers a direct, unprocessed view of the GSR data.
- (b) Phasic GSR Signal: This graph shows the phasic component of the GSR signal, resulting from a filtering process. The local average for each GSR measurement (within an interval of ±4 s) was calculated, and this average was subtracted from the original GSR values. The resulting signal highlights the rapid, short-term fluctuations (phasic responses), providing insights into the subject’s immediate stress reactions. This was generated using Python 3.10 code in Google Colab.
- (c) Highlighting GSR Peaks and Mean Value: This graph identifies the key peaks in the GSR signal and highlights the mean GSR value (3.25 µS in this example). Peaks are marked with red crosses, helping to emphasize moments of heightened stress response. This visualization gives a clearer view of the subject’s stress levels over time, focusing on deviations from the average. It was created using Python to automate the peak detection and analysis.
- (d) Combined GSR and Phasic Signal: This graph combines the original GSR signal with the phasic GSR signal, providing a comparative view of both datasets. The blue line represents the raw GSR values, while the orange line depicts the phasic GSR values. This allows for a comprehensive analysis of both the subject’s overall stress response and the moment-to-moment changes. The graph was produced using the same Python code as the second plot.

### 3.2. Machine Learning Analysis of GSR Data

To enhance the analysis of GSR sensor data, machine learning techniques were applied using data from YAAD and eSports datasets.

**Figure 6:**
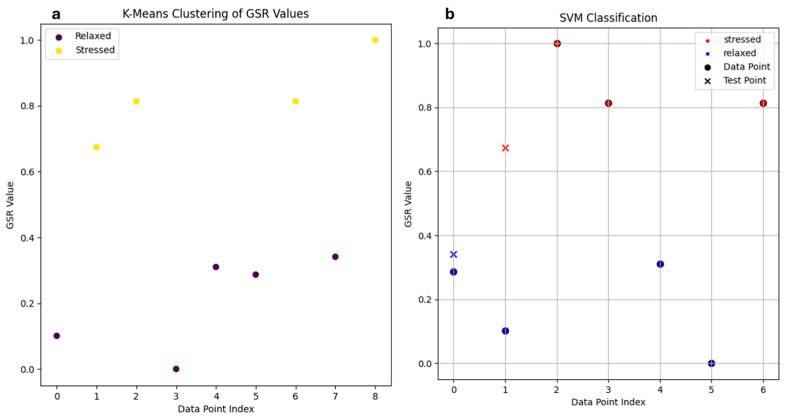
GSR data classification; (**a**) result of applying a K-means clustering algorithm to the GSR data. Yellow indicates subjects who showed elevated levels of anxiety. In purple are those who showed no noticeable change in GSR data; (**b**) classification results with SVM algorithm. The circle represents the training set data while the symbol “x” represents the test set data. The data categorized as unstressed are represented in blue while the test set categorized as stressed are represented in red.

Figure 6a shows the results of applying a K-means clustering algorithm to the GSR data, which separates the participants into two categories: those experiencing noticeable stress (represented in yellow) and those who remain relatively calm (shown in purple). The vertical axis represents the normalized GSR values, while the horizontal axis represents individual data points. The clustering method efficiently grouped the subjects based on their GSR response during the diagnostic testing process.

Clustering assigns labels (stressed, relaxed) to the data. After the data has been labeled, the SVM algorithm can be applied to them, resulting in a supervised learning process. Figure 6b displays the classification results, where the training set data are represented as circles and test set data as “x” markers. Blue indicates the unstressed individuals, while red marks those categorized as stressed. This approach demonstrated that GSR peak amplitude values could be effectively classified with a linear decision boundary, highlighting the separability of GSR patterns in relation to stress. However, in some cases, more complex data may require nonlinear decision boundaries for more accurate classification.

In that case, the model’s accuracy reached 100%. This was expected, mainly due to the small sample size and the fact that the GSR peak amplitude values were easily separable. The two test set elements can be separated in a linear fashion. However, as the dataset size increases, it is more likely that the data points will become more difficult to classify into two distinct classes with a simple linear decision boundary. As a result, misclassification of test samples may occur, and some training samples, such as stressed individuals, could fall into regions where the SVM model classifies them as unstressed. This would naturally lead to a reduction in the model’s overall accuracy.

**Figure 7:**
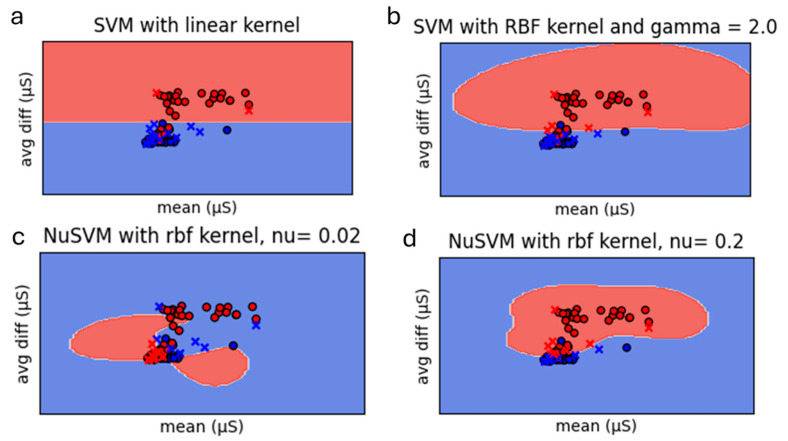
SVM Classification; (**a**) using a linear kernel; (**b**) using Radial Basis Function (RBF) Kernel with gamma value equal to 2.0; (**c**) using NuSVM with RBF kernel and ν = 0.02; (**d**) using NuSVM with RBF kernel and ν = 0.2.

Figure 7 illustrates the further analysis that was conducted using both the linear and Radial Basis Function (RBF) kernels, employing the mean value of the GSR signal and the difference between the GSR peak amplitude and the mean value. Figure 7a shows the SVM algorithm with a linear kernel. This method, that attempted to separate the samples by constructing a linear decision boundary, demonstrated a clear linear decision boundary, as expected for linearly separable data. However, this approach had limitations when applied to more complex datasets. Figure 7b shows the RBF kernel with the gamma parameter set to 2.0. The gamma value plays a crucial role in shaping the decision boundary and determining its complexity, since lower gamma values lead to broader Gaussian functions, resulting in smoother and less intricate decision boundaries, while higher gamma values produce narrower Gaussian functions, yielding more complex and finely detailed decision boundaries. The results showed that the decision boundary obtained using the RBF kernel differed significantly from the linear method. By transforming the input data into a higher-dimensional space, the RBF kernel effectively leveraged the proximity of data points in this new space to classify samples with improved accuracy.

Building upon the initial analysis using standard SVM with linear and RBF kernels, additional classification experiments were conducted using the NuSVM algorithm. This approach incorporates the parameter ν, which ranges from 0 to 1, to control the trade-off between the number of support vectors and the tolerance for misclassification errors. Lower values of ν allow for more misclassifications and increase the number of support vectors, while higher values restrict the margin size and reduce support vectors. Two different values of ν were tested: ν = 0.02 (Figure 7c) and ν = 0.2 (Figure 7d). The RBF kernel was utilized to transform the GSR data into a higher-dimensional space, allowing for the modeling of complex, nonlinear decision boundaries.

The evaluation of the models revealed notable differences between the performance of the linear kernel and the RBF kernel. For the linear kernel, the stress classification results showed that 11.11% of the samples were categorized as stressed, while 88.89% were classified as unstressed. The model achieved an accuracy score of 40% and an F1 score of 25%. The significant discrepancy between these two scores indicated overfitting, as the model disproportionately favored the unstressed class over the stressed class. In contrast, the RBF kernel demonstrated improved performance. The stress classification results revealed that 27.78% of the samples were identified as stressed, and 72.22% as unstressed. This model achieved an accuracy score of 50% and an F1 score of 44.44%. The closeness of the accuracy and F1 scores suggested that the RBF model provided better generalization and avoided overfitting, making it more effective for this classification task.

To further improve classification performance, the NuSVM algorithm was employed. The NuSVM model with ν = 0.02 classified 61.11% of the samples as stressed and 38.89% as unstressed. This configuration achieved an accuracy score of 60% and an F1 score of 75%. These values indicate that the model performed effectively, with a high level of balance between precision and recall.

The results for ν = 0.2 show a balanced classification: 50% of the samples were categorized as stressed, and 50% as unstressed. This configuration improved the overall performance, achieving an accuracy score of 70% and an F1 score of 72.73%. The minimal difference between accuracy and F1 scores suggests strong generalization and a balanced performance across stress classification categories.

The NuSVM algorithm demonstrated improved performance compared to the standard SVM configurations. The model with ν = 0.2 showed the best balance between accuracy and F1 score, making it the most suitable configuration for this dataset. These findings highlight the advantage of employing NuSVM for handling complex datasets like GSR signals, where a flexible trade-off between margin size and misclassification tolerance can optimize performance.The RBF kernel outperformed the linear kernel, achieving higher accuracy and F1 scores. This outcome aligns with expectations, as real-world experimental data rarely exhibit perfect linear separability. The RBF kernel’s capacity to adapt to complex data distributions makes it more suitable for stress classification in GSR data.

## 4. Discussion

From a sociological perspective, screening is not only a medical intervention but also a social one, raising important questions about its implementation and the information provided to the public. According to Armstrong and Eborall (2012), screening creates a significant market, and the data about its efficacy and limitations are not always clearly presented to maximize benefits. This can lead to misuse and false certainty about one’s health status [17].

During the COVID-19 pandemic, testing became ubiquitous and dispersed from laboratory and healthcare settings to reach individuals’ homes with mass self-testing. While from early on the use of RT-PCR to detect SARS-CoV-2 was adopted mostly for clinical use, its use broadened. However, for screening purposes during the pandemic, testing capacity and cost, as well as the time needed to yield a result (a timely diagnosis), are all crucial factors for public health interventions. The rapid antigen tests became available for mass use and are low cost, while they can provide quick results (up to 30 min). Compared to PCR tests, rapid tests demonstrate poorer sensitivity, meaning that (depending on the manufacturer) they can have false-negative results. In the case of COVID-19, the rapid tests might not detect an asymptomatic individual and provide a false re-assurance on one’s status. Whereas, their sensitivity is higher when used in the onset of the disease symptoms when an individual has a high viral load. Rapid tests have a high specificity (rarely produce false positives). Given their advantages, states variously employed mass testing strategies with rapid tests during the pandemic.

Two issues are of importance here. The mass use of rapid tests occurred beyond the healthcare settings, either at designated screening sites (usually where healthcare professionals are involved) or at home (by an individual bearing responsibility to undertake the test). The second issue is the fact that rapid tests do not offer diagnostic certainty, with implications for an individual becoming responsible to judge upon uncertainty [18].

In the case of Greece, where the research was undertaken, Rapid tests were part of the strategy to manage COVID-19 pandemic while restoring economic, educational and, in general, social life. In the third phase of the pandemic rapid tests were performed by the public health authorities (free, voluntary, random screening) while groups of citizens were entitled to free tests (or needed to buy them) to use at-home and then report the result. An additional testing site was pharmacies, where citizens could pay a pharmacist to perform the test and report the result to the authorities. Reporting a negative result was a prerequisite to participate in educational activities and to go to work. Reporting a result in the interconnected registries, for the employees connected to the one of social security, was done through a designated website. This research was conducted in pharmacies, meaning beyond a healthcare setting. As such, its results are of great significance regarding the employment of screening practices without direct guidance by a healthcare professional and with a heightened responsibility assumed by the individuals. In addition, as similar approaches of mass testing with self-tests could be expanded in future screening practices, the uncertainties involved are of great importance.

Economically, while screening is less expensive on an individual basis, it becomes costly when applied to an entire population. Hence, screening is often recommended for specific high-risk groups. This approach is based on statistical risk factors rather than certainties, which can lead to misunderstandings and stereotypes if not communicated transparently. Limiting screening to certain groups should be conducted with a clear explanation to avoid misconceptions about who is at risk.

Psychologically, screening can induce anxiety, especially when results indicate a need for further examination, and can create false reassurance from negative results despite low sensitivity and specificity [19,20]. Participation in screening is critical, as it involves a large number of asymptomatic people to identify those at immediate risk. High attendance increases the effectiveness of screening in reducing health risks within a population. However, several factors influence participation rates, including comfort with being monitored, sense of civic responsibility, awareness, and the potential stress and fear associated with false diagnoses.

These factors can deter individuals from participating, thereby reducing the overall efficiency of screening programs. Armstrong and Eborall emphasize the need to look beyond individual experiences and examine the infrastructure of screening technologies, their design, implementation, and impact [17]. Ensuring clear communication and addressing the psychological and sociological aspects of screening can enhance participation and, consequently, the effectiveness of these programs. Understanding and addressing the stress and fear associated with screening is essential. When people are apprehensive about participating due to stress and fear of potential outcomes, participation rates drop, undermining the efficiency of screening programs. Efforts to educate the public, reduce anxiety, and make the screening process as transparent and reassuring as possible are critical to improving participation and the overall success of these health interventions.

While diagnostic testing is a cornerstone in managing SARS-CoV-2 and other viral diseases, it also involves significant psychosocial dimensions at the level of the individual. In fact, diagnostic testing may typically provoke feelings of anxiety and stress among patients, based on fear of the positive result, uncertainty of a test outcome, and larger implications for personal and social responsibilities.

The ways to measure stress in the human body proposed by the scientific community are heart rate measurement (HR) [21], observation of facial expressions [22], GSR [23], finger temperature (FT) [24], pupil dilation eye tracking (PD), as well as eye tracking (ET) [25] and other methods. Among all the above alternatives, the study with a sensor that measures the GSR was chosen. The hypothesis that anxiety affects participation in diagnostic tests (rapid test) for COVID-19 was investigated and confirmed. The level of anxiety was measured and recorded in an objective manner. To achieve this, a sensor (connected to a microcontroller) was used, and the GSR was measured. Based on the tests performed and the corresponding graphs obtained, the cases of subjects studied, it was established, from the comparative study of the measurement curves, that they could be grouped into three (3) basic categories.

- 1st Category: it is observed that 36 out of 51, i.e., 71%, noted an increase in anxiety/stress levels during the rapid test for the COVID-19 pandemic.
- 2nd Category: in a percentage of 24%, there was no increase in stress levels during diagnostic testing for the COVID-19 pandemic, as there was no particular change in GSR values during the rapid test and therefore they were in a tranquility state.
- 3rd Category: In a percentage of 5% valid measurements were not performed, due to some experimental error (e.g., poor contact of electrodes on the skin). This category of measurements shows values outside the normal expected limits of the GSR. These limits are from 1 to 20 µS. Therefore, if in a test there is no good contact of the sensor with the subject’s fingers, then the value corresponding to air measurements is measured, i.e., below 1 μS (outside normal limits) and specifically 0.93 μS.

While the measurements were taken in the third phase of the pandemic, when most people were now familiar with the diagnostic tests, a relative increase in GSR values is still observed, corresponding to an increase in stress. Therefore, even though today stress may affect a person less than in the first phase of the pandemic, as can be seen in the graphs of the measurements, it remains a significant factor that may reduce individual participation in screening strategies. After all, similar findings have also been recorded for the anxiety experienced by the subjects of various medical examinations and measurements, either due to the measurement process itself, or due to the criticality of the results (e.g., cardiac examinations, blood pressure measurement, etc.).

Machine learning techniques were applied to improve the analysis of GSR data. K-means clustering successfully grouped the individuals based on their GSR responses, while the SVM algorithm demonstrated high accuracy in classifying stress levels, achieving a perfect classification in this study’s dataset. This high accuracy is attributed to the dataset’s simplicity and small sample size, which allowed for clear separation between the classes. The classification results indicate that GSR peak amplitudes, under the conditions studied, were linearly separable, allowing the SVM model to achieve excellent classification performance.

The findings from this study suggest that machine learning approaches can significantly enhance the analysis of physiological data like GSR for stress detection. By applying unsupervised clustering followed by supervised classification, it is possible to identify stress states with higher precision than traditional methods. These results support the potential for machine learning algorithms to be integrated into real-time stress monitoring systems, providing objective assessments in clinical, occupational, or high-performance environments. Furthermore, this study adds to the growing body of research demonstrating the utility of GSR in stress detection, particularly when combined with advanced analytical techniques.

While our study demonstrates the effectiveness of the RBF kernel in classifying stress states, previous research has shown that nonlinear models may be prone to overfitting, particularly when working with small datasets [26]. Further research emphasizes the significance of addressing computational complexity and the challenges of parameter tuning associated with nonlinear kernels [27]. To overcome these limitations, future studies should focus on incorporating larger datasets and implementing advanced techniques for hyperparameter optimization

Potential confounders, such as participants’ familiarity with testing procedures, baseline stress levels, and environmental factors, were not controlled in this study. A pre-test survey assessing participants’ prior exposure to rapid tests and baseline stress levels could enhance future studies by accounting for these variables.

The study’s sample size of 51 participants limits the generalizability of the findings. Additionally, demographic data such as age, gender, and medical history, which could influence stress responses, were not included. Future studies should focus on larger, more diverse participant groups and stratify data by demographics to allow for subgroup analysis and improve the applicability of the findings.

## 5. Conclusions

This study confirmed that undergoing COVID-19 rapid tests is associated with a noticeable increase in stress levels, as measured by a GSR sensor. The collected data from 51 participants highlighted that many individuals experienced heightened anxiety during the testing process, validating the hypothesis that diagnostic procedures can trigger stress responses. By incorporating machine learning techniques, specifically clustering and classification algorithms, the analysis of GSR data was enhanced. These algorithms effectively distinguished between “stressed” and “not stressed” participants, demonstrating the potential of automated classification in the context of physiological stress monitoring. These findings underscore the importance of considering psychological factors in public health screening processes. Addressing anxiety could be key to improving participation rates and ensuring the effectiveness of future diagnostic campaigns, emphasizing the need for patient-centered approaches in healthcare settings.

## Data Availability

The raw data supporting the conclusions of this study are available on request from the corresponding author. The data are not publicly available due to ethical considerations, as they involve anonymized physiological measurements from human participants. Sharing the data requires ensuring compliance with privacy and ethical guidelines.
